# Using machine learning methods to investigate the impact of comorbidities and clinical indicators on the mortality rate of COVID-19

**DOI:** 10.3389/fmedt.2025.1621158

**Published:** 2025-09-22

**Authors:** Yueh-Chen Hsieh, Sin Chen, Shu-Yu Tsao, Jiun-Ruey Hu, Wan-Ting Hsu, Chien-Chang Lee

**Affiliations:** ^1^ Department of Laboratory Medicine, National Taiwan University Hospital Yunlin Branch, Douliou, Taiwan; ^2^ Department of Medicine, College of Medicine, National Taiwan University, Taipei, Taiwan; ^3^ Department of Emergency Medicine, National Taiwan University Hospital, Taipei, Taiwan; ^4^ Department of Cardiology, Smidt Heart Institute, Cedars-Sinai Medical Center, Los Angeles, CA, United States; ^5^ Department of Epidemiology, Harvard T.H. Chan School of Public Health, Boston, MA, United States; ^6^ Clinical AI Consulting Group, Brookline, MA, United States; ^7^ Department of Information Management, Ministry of Health and Welfare, Taipei, Taiwan

**Keywords:** COVID-19, mortality, survival analysis, machine learning, federated learning

## Abstract

**Background:**

This study aims to develop a machine learning model to predict the 30-day mortality risk of hospitalized COVID-19 patients while leveraging federated learning to enhance data privacy and expand the model's applicability. Additionally, SHapley Additive exPlanations (SHAP) values were utilized to assess the impact of comorbidities on mortality.

**Methods:**

A retrospective analysis was conducted on 6,321 clinical records of hospitalized COVID-19 patients between January 2021 and October 2022. After excluding cases involving patients under 18 years of age and non-Omicron infections, a total of 4,081 records were analyzed. Key features included three demographic data, six vital signs at admission, and 79 underlying comorbidities. Four machine learning models were compared, including Lasso, Random Forest, XGBoost, and TabNet, with XGBoost demonstrating superior performance. Federated learning was implemented to enable collaborative model training across multiple medical institutions while maintaining data security. SHAP values were applied to interpret the contribution of each comorbidity to the model's predictions.

**Results:**

A subset of 2,156 records from the Taipei branch was used to evaluate model performance. XGBoost achieved the highest AUC of 0.96 and a sensitivity of 0.94. Two versions of the XGBoost model were trained: one incorporating vital signs, suitable for emergency room applications where patients come in with unstable vital signs, and another excluding vital signs, optimized for outpatient settings where we encounter patients with multiple comorbidities. After implementing federated learning, the AUC of the Taipei cohort decreased to 0.90, while the performance of other cohorts improved to meet the required standards. SHAP analysis identified comorbidities including diabetes mellitus, cerebrovascular disease, and chronic lung disease to have a neutral or even protective association with 30-day mortality.

**Conclusion:**

XGBoost outperformed other models making it a viable tool for both emergency and outpatient settings. The study underscores the importance of chronic disease assessment in predicting COVID-19 mortality, revealing some comorbidities such as diabetes mellitus, cerebrovascular disease and chronic lung disease to have protective association with 30-day mortality. These findings suggest potential refinements in current treatment guidelines, particularly concerning high-risk conditions. The integration of federated learning further enhances the model's clinical applicability while preserving patient privacy.

## 1 Introduction

Coronavirus disease 2019 (COVID-19) is a contagious disease caused by the virus SARS-CoV-2, which has had a profound impact on global economies, healthcare systems, and social norms (1). Since the initial case was identified in Wuhan, China, in December 2019 (2), over 777 million individuals have been infected, and more than 7 million have died worldwide (as of 9 February 2025) (3). The average time from exposure to symptom onset is five days, and approximately 5% of patients with COVID-19 experience severe symptoms necessitating intensive care (4). While the diagnosis of COVID-19 is facilitated by the use of rapid antigen tests (RATs) (5) and polymerase chain reaction (PCR) (6) technology, the challenge lies in accurately assessing the severity of the disease based on clinical data and chest x-ray features (7). In 2020 WHO developed a Clinical Progression Scale, patients have been categorized as those with mild disease (ambulatory, not requiring supplemental oxygen), those with moderate disease (hospitalized, might requiring low-flow oxygen), and those with severe COVID-19 (on HFNC, NIV, IMV, or ECMO) (8). Nevertheless, accurate prediction of the prognosis of COVID-19 remains an elusive endeavor. Predicting COVID-19 mortality is important as it has significant implications for the selection of pharmacologic treatments, management strategies, and for family planning and goals of care discussions (9).

Previous clinical decision models have focused on common health data rather than comorbidities, which might have negative impact on accuracy since comorbidities and COVID-19 mortality are correlated (10). The 4C Mortality Score, which was identified as the most promising risk stratification model in numerous systematic reviews (11, 12), is a risk stratification tool that predicts in-hospital mortality rate for hospitalized COVID-19 patients with eight parameters (age, sex, number of comorbidities, respiratory rate, peripheral oxygen saturation, level of consciousness, urea level, and C reactive protein) (13). Risk stratification tool is a method that predict one's risk based on its clinical histories and other factors. The 4C mortality score sum up the scores of the eight parameters and range from 1 to 21, each represent a certain mortality rate respectively. While the 4C Score trained with 35,463 patients showed moderate diagnostic accuracy for mortality with derivation cohort area under the receiver operating characteristic curve (AUC) of 0.79, it performs poorly on other cohorts, with AUC ranging from 0.63 to 0.73 (13). In the present study, we sought to increase the accuracy by incorporating the identity of the comorbidity, not just the number of comorbidities, to the prediction. We developed the Comorbidities and Clinical Indicators on the Mortality Model (CCIMM), a machine learning model that can accurately predict a patient's mortality rate within 30 days of hospitalization, using 79 comorbidities that are readily available at the time of admission.

## 2 Methods

### 2.1 Data collection

National Taiwan University Hospital (NTUH) operates three major branches in Taipei, Hsinchu, and Yu nlin, all of which are tertiary medical centers, with 2,600 beds, 1,500 beds, and 900 beds, respectively. This study collected clinical data retrospectively on patients hospitalized with Omicron variant COVID-19 from April 2022 to October 2022 across the three hospitals. Data included demographics, admission vital signs [e.g., temperature, breath rate, pulse rate, systolic blood pressure [SBP], diastolic blood pressure [DBP]], and underlying comorbidities. This research project was approved by the ethics committee of National Taiwan University Hospital Institutional Review Board. The study was conducted in accordance with the principles of the Declaration of Helsinki and the Good Clinical Practice Guidelines, and all the participants were informed consent. We have no access to information that could identify individual participants during or after data collection.

### 2.2 Outcomes

The primary outcomes were 30-day all-cause mortality. Mortality outcomes were verified by linking the database with the national death registry, enabling accurate determination of survival status after discharge. Institutional review boards of each respective hospital approved waivers of informed consent, and all data were deidentified.

### 2.3 Missing data

The *missForest* R package was used to impute missing values for continuous variables such as age, body mass index (BMI), temperature, respiratory rate, pulse rate, SBP, and DBP. This iterative method constructs random forest models for each variable with missing data, using observed values from other variables as predictors to estimate the missing values.

### 2.4 Input variables

Two sets of data were subjected to the establishment of the model: one comprising demographic information, vital signs upon admission, and underlying comorbidities; the other, comprising only demographic information and underlying comorbidities. The datasets include 79 comorbidities and 6 vital signs. A *p*-value < 0.05 in comorbidities and vital signs was considered to be statistically significant between COVID-19 survivors and non-survivors. A total of 6,321 patients were identified across the three hospitals, with 2,240 excluded due to patient age under 18 or the identification of non-Omicron variants of SARS-CoV-2. The discovery of Omicron variants was in South Africa in late November 2021 (14). In Taiwan, the Omicron variant became predominant in April 2022, causing the second epidemiological surge (15). We only use data after April 2022 to stratified Omicron variants. The Taipei cohort, comprising 2,156 patients, was employed for the model development. The Hsinchu and Yunlin cohorts were utilized in federated learning.

### 2.5 Machine learning methods

The dataset was divided into training and validation subsets, with 70% allocated for training and 30% for validation. To address the issue of class imbalance in case outcomes, the Synthetic Minority Oversampling Technique (SMOTE) was applied, enabling the oversampling of minority-class patients within the training dataset. Four machine learning models, including Lasso (16), Random Forest (17), TabNet (18), and Gradient-Boosted Tree (XGBoost) (19), were utilized. The models were implemented using the following Python libraries: *sklearn* (LogisticRegression and RandomForestClassifier), *pytorch-tabnet*, and *xgboost* (version 2.3.1).

Random Forest and XgBoost are ensemble learning methods that combine multiple weak decision trees to generate a strong decision tree. Random Forest uses the “bagging” method, where each tree is trained on a bootstrap sample of the data, and their predictions are aggregated by voting in classification tasks or averaging in regression tasks. The trees are trained independently and do not update each other, making the method relatively fast and robust against overfitting (17). In contrast, XGBoost is a gradient boosting method which builds trees sequentially, where each new tree focuses on correcting the residual errors of the previous trees using gradient information. The final prediction is obtained by summing the weighted outputs of all trees. This sequential learning allows for effective error correction and high accuracy but may increase the risk of overfitting if not properly regularized (19).

Lasso regression is a type of linear regression that adds a penalty term to reduce the size of the model's coefficients, helping to prevent overfitting. Although the model remains linear, the added penalty can shrink some coefficients exactly to zero, removing less useful features from the model. This results in a simpler, more interpretable model that often performs better on data with unrelated features (16).

Finally, TabNet is a method designed for tabular data. Its advantage lies in its ability to provide feature-level interpretability through attention masks. However, its performance can be sensitive to hyperparameter tuning, and training the model can be computationally demanding (18).

Shapley Additive Explanations (SHAP) were employed to enhance the interpretability of these models, allowing the quantification of each predictor's contribution to the model's predictions (20). Additionally, an unsupervised clustering approach based on Euclidean distance was used to group patients with similar SHAP profiles, aiding in the identification of phenotypic patterns within the study cohort.

### 2.6 Federated learning

Federated Learning (FL) was implemented to integrate the most effective model from the Taipei cohort with additional data from Hsinchu and Yunlin hospitals. FL is a decentralized and collaborative approach designed to address challenges related to data silos and sensitivity (21). Since the datasets from these hospitals shared identical features but varied in sample composition, horizontal alliance learning was employed. The process began with each participating hospital receiving the same initial model and parameters. Each hospital independently trained the model on its local data, computed gradient updates, and securely transmitted these updates to a central server. The server aggregated the encrypted gradients, updated the global model, and redistributed the improved model parameters back to the hospitals. This iterative process allowed the final model to generalize across datasets while preserving data privacy, enabling its application to a broader population.

### 2.7 Performance evaluation

The study aimed to predict 30-day mortality by employing a comprehensive set of evaluation metrics to assess model performance. The metrics included measures to capture predictive accuracy, precision, and reliability. Additionally, distinctions in vital pathogenic factors were evaluated across subgroups defined by gender, age, and body mass index (BMI). Gender was categorized into male and female groups, while age was segmented into elderly (≥65 years), middle-aged (40–64 years), and young (18–39 years) groups. BMI was classified into obese (≥30), overweight (25–29), and normal (<25) categories. These subgroup analyses provided insights into model performance variations across diverse patient demographics and clinical characteristics. All computational analyses were conducted using Python 3.10.8.

## 3 Results

### 3.1 Participants

**Figure 1:**
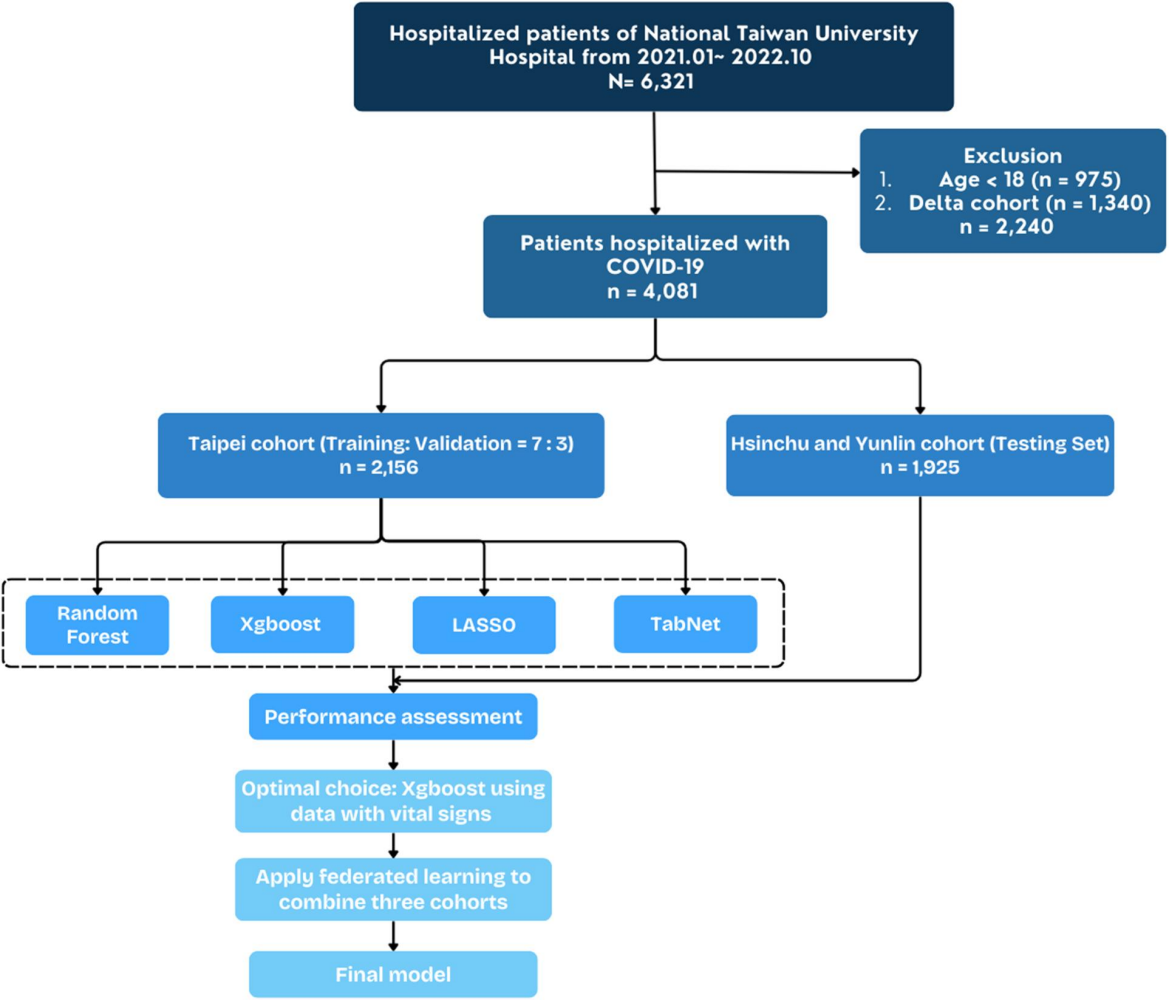
Flowchart depicting construction of study cohort and external validation cohort from national Taiwan university hospital cohort.

The study included 4,081 hospitalized adult patients diagnosed with SARS-CoV-2 Omicron variant infections between April 2022 and October 2022, with the diagnosis confirmed by real-time polymerase chain reaction (RT-PCR). The initial registry consisted of 6,321 patients hospitalized for COVID-19 across three branches of NTUH—Taipei, Hsinchu, and Yunlin—from January 2021 to October 2022. Figure 1 illustrates the patient selection flowchart. Table 1 summarizes the demographic information, vital signs upon admission, and underlying comorbidities stratified by primary outcome. Among the final cohort, 2,015 patients survived, while 141 patients succumbed to the disease. Non-survivors were significantly older than survivors, with a median age of 78 years compared to 69 years (*p* < 0.001). Apart from temperature, vital signs differed significantly between survivors and non-survivors (*p* < 0.001). Chronic kidney disease and metastatic cancer were significantly associated with mortality (*p* < 0.001).

**Table 1:** Characteristics of COVID patients in the Taipei cohort, stratified by 30-day survival status.

Characteristic	COVID-19 survivors (*n* = 2,015)	COVID-19 non-survivors (*n* = 141)	Total (*n* = 2,156)	*P*-value
Age, mean (SD)	69 (19)	78 (16)	70 (19)	< 0.001
Male gender, *n* (%)	1,050 (52.1%)	75 (53.2%)	1,125 (52.2%)	0.872
BMI, mean (SD)	22.81 (4.77)	22.83 (4.09)	22.81 (4.72)	0.967
BMI >30, *n* (%)	121 (6%)	6 (4.3%)	127 (5.9%)	0.504
Vital sign				
Temperature (SD)	36.7 (0.66)	36.5 (0.61)	36.7 (0.66)	0.001
Breath rate (SD)	19 (3.19)	21 (4.92)	19 (3.35)	< 0.001
Pulse rate (SD)	88 (16.72)	94 (20.53)	88.24 (17.05)	< 0.001
SBP (SD)	136 (22.56)	128 (29.21)	135 (23.03)	< 0.001
DBP (SD)	77 (12.59)	73 (16.62)	76 (12.86)	< 0.001
SpO2	97 (1.97)	96 (4.34)	97 (2.24)	< 0.001
Underlying comorbidity				
Hypertension (%)	1,115 (55.3%)	96 (68.1%)	1,211 (56%)	0.004
Chronic lung disease (%)	223 (11.1%)	9 (6.4%)	32 (10.8%)	0.111
Chronic kidney disease (%)	526 (26.1%)	63 (44.7%)	589 (27.3%)	< 0.001
Cancer without metastasis (%)	559 (27.7%)	57 (40.4%)	616 (28.6%)	0.002
Metastatic cancer (%)	193 (9.6%)	31 (22%)	224 (10.4%)	< 0.001
Diabetes mellitus (%)	624 (31%)	52 (36.9%)	676 (31.4%)	0.171
Peptic ulcer disease (%)	173 (8.6%)	16 (11.3%)	189 (8.8%)	0.333
Prior use of steroid (%)	436 (21.6%)	37 (26.2%)	473 (21.9%)	0.241

CNS, central nervous system; SE, standard error.

### 3.2 Mortality prediction in machine learning models

The Taipei cohort was used to train and validate a machine learning model, while the Hsinchu and Yunlin branches implemented federated learning to evaluate generalizability. Demographic information, vital signs, and comorbidities were used to predict 30-day mortality. Table 2 compares the performance of machine learning algorithms, with Xgboost achieving the highest area under the curve (AUC), area under the precision-recall curve (AUPRC), sensitivity, and negative predictive value (NPV) when specificity was set at 0.80. The DeLong test is a nonparametric statistical method used to compare AUCs of models that are tested on the same dataset, and it provides a *p*-value to determine whether the difference between the two AUCs is statistically significant (22). Table 3 uses DeLong test to compare Xgboost with other algorithms. *P*-value < 0.05 means that Xgboost and that algorithms has significant difference, which also indicates that Xgboost performs better.

**Table 2:** Measures of model discrimination and accuracy in the validation dataset (NIS 2014)sss, including area under the curve (AUC) and its 95% confidence intervals, sensitivity, specificity, positive predictive value (PPV), and negative predictive value (NPV).

Model	AUC	AUPRC	Sensitivity	Specificity	PPV	NPV
Lasso	0.84 (95% CI 0.829–0.860)	0.70	0.71	0.80	0.52	0.91
Random forest	0.92 (95% CI 0.919–0.928)	0.83	0.81	0.80	0.67	0.94
Xgboost	0.96 (95% CI 0.967–0.974)	0.83	0.94	0.80	0.57	0.97
TabNet	0.78 (95% CI 0.752–0.804	0.68	0.60	0.80	0.46	0.88

**Table 3:** Delong test results comparing the AUC of XGBoost against Lasso, Random Forest, and TabNet models.

Model 1	Model 2	ΔAUC	*p*-value
XGBoost	Lasso	0.12	< 0.001
XGBoost	Random Forest	0.04	< 0.001
XGBoost	TabNet	0.18	< 0.001

ΔAUC represents the difference in AUC (XGBoost−other model), and *p*-values indicate the statistical significance of the difference.

The inclusion of vital signs enhanced the Xgboost model's performance compared to using only demographic information and chronic illnesses. Table 4 shows that incorporating vital signs improved all performance metrics, including AUC, sensitivity, and NPV. The results highlight the importance of vital signs in achieving superior predictive accuracy. In conclusion, the Xgboost model was the most effective prediction tool for 30-day mortality, offering a robust balance between sensitivity and specificity while maintaining a conservative approach.

**Table 4:** Xgboost model using different sets of features.

Model	AUC	AUPRC	Sensitivity	Specificity	PPV	NPV
Vital signs and chronic illness	0.96	0.83	0.94	0.80	0.57	0.97
Chronic illness alone	0.93	0.72	0.88	0.80	0.54	0.96

### 3.3 Feature importance and SHAP analysis

**Figure 2:**
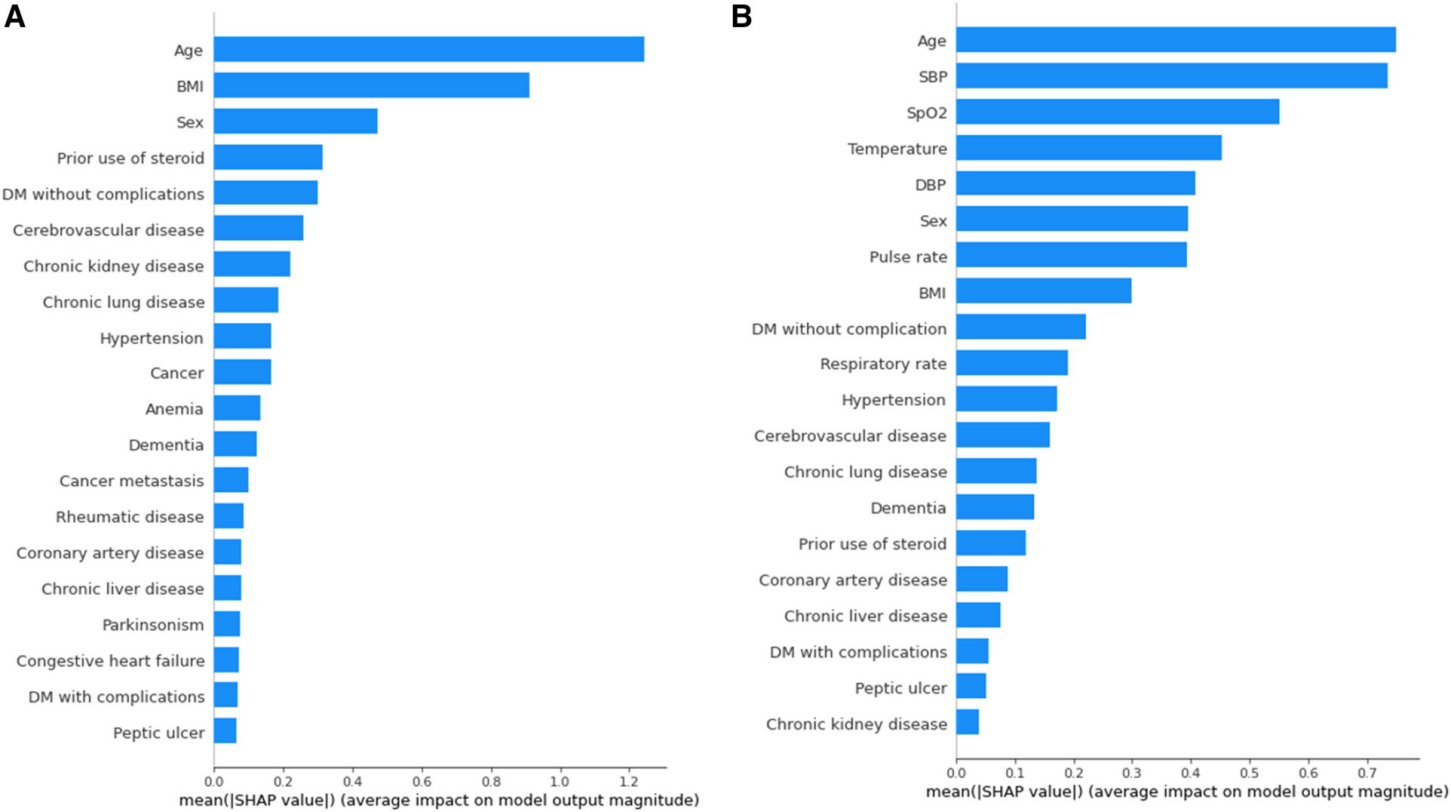
Variables of importance (20 most important variables) from xgboost, ranked by mean SHAP values. **(A)** Model using both vital and chronic illness. **(B)** Model using only chronic illness.

**Figure 3:**
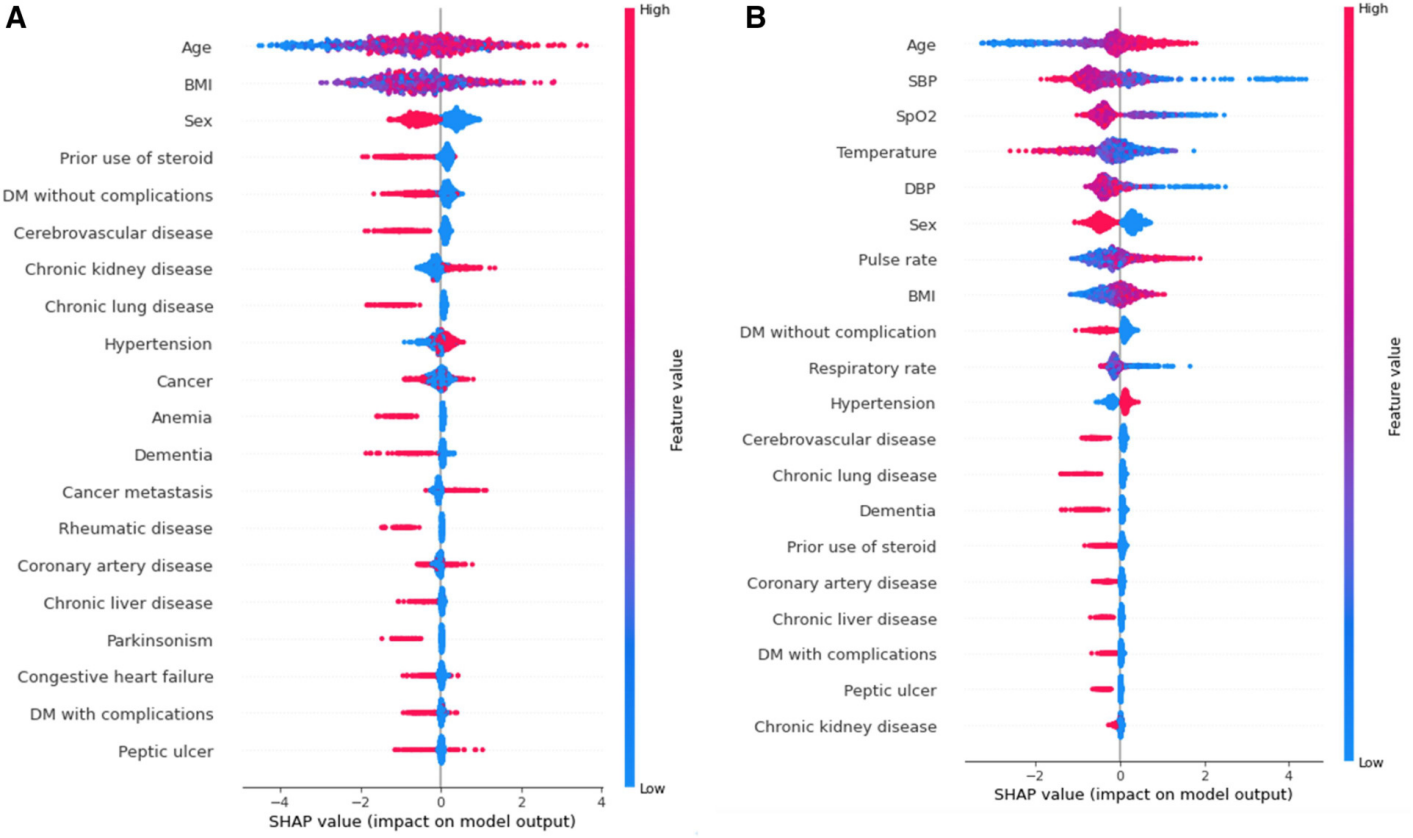
Bee swarm plot valuing feature impact on predictions, where red and blue dots represent high and low feature values, respectively. Overlapping dots indicate instability without vital signs, highlighting their importance for stable and accurate predictions. **(A)** Model using both vital and chronic illness. **(B)** Model using only chronic illness.

To provide a visual representation of the selected features, the SHAP approach was employed to illustrate their impact on 30-day mortality in the Xgboost model (23). The mean SHAP value plot (Figure 2) ranks the top 20 features with the highest average absolute SHAP values, while the bee swarm plot (Figure 3) presents the individual contributions of these features, offering insights into their stability and interpretation. In both figures, feature rankings indicate their importance to the predictive model, while SHAP values provide a unified measure of the influence of specific features. In the bee swarm plot, red dots represent high feature values, while blue dots indicate low values, enabling visualization of how each feature affects predictions.

Certain features, including age, pulse rate, and body mass index (BMI), are associated with an increased risk of mortality when their values are elevated. Conversely, lower values of systolic blood pressure (SBP), oxygen saturation (SpO₂), temperature, diastolic blood pressure (DBP), and respiratory rate are linked to higher mortality risk. Chronic illnesses such as diabetes mellitus without complications, cerebrovascular disease, and chronic lung disease are also significant predictors, but some of these features, surprisingly, are associated with a lower likelihood of mortality. This trend highlights different correlations between features(demographic information, vital signs, comorbidities) and mortality rate.

The mean SHAP value plot (Figure 2) confirms the importance of both vital signs and chronic illnesses, with features such as age, BMI, and sex emerging as the most critical contributors. In the absence of vital signs, the bee swarm plot (Figure 3B) shows overlapping red and blue dots, indicating instability, as the same feature value can exert different effects on prediction (23, 24). Without vital signs, the SHAP values of chronic illnesses, including prior steroid use, cerebrovascular disease, and chronic kidney disease, dominate the model. However, the decline in SHAP values for most features suggests that the model is less robust and lacks sufficient influential factors (25, 26).

The inclusion of vital signs in the model improves both stability and predictive accuracy, with six vital signs ranking among the top ten features. The absence of vital signs reduces model stability and precision, particularly for certain subgroups. For instance, stratified analysis (Table 5) reveals that predictions for individuals with a BMI of 30 or higher are less precise. Consequently, BMI ≥ 30 is considered outside the scope of the model. These results underscore the critical role of vital signs in achieving reliable and stable predictions, while also identifying the chronic illnesses that have the most significant impact when vital signs are excluded.

**Table 5:** The area under the curve (AUC) in different subgroups gender (male), Age (elderly >= 65 y/o., middle age 40–64 y/o, young 18–39 y/o), BMI (obese >=30, overweight 25–29, normal <25).

Groupings	Subgroups	AUC
All patients	All patients	0.96
Sex	Male	0.98
	Female	0.97
Age	Age ≥65	0.97
	65> Age ≥40	1.00
	39 > Age ≥18	1.00
BMI	BMI ≥30	0.87
	30 > BMI ≥25	0.97
	BMI <25	0.98

### 3.4 Model performance and federated learning

Federated learning is a method where individuals, such as hospitals, can train their data on a local end and upload the parameters to the head server. This can prevent data leakage and transmission of large quantity data. We use Federated learning to combine training results from three hospital branches- Taipei, Hsinchu and Yunlin.

**Figure 4:**
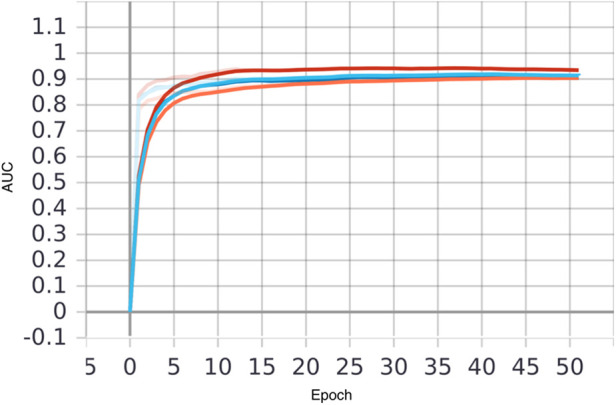
Comparison of AUC of local and federated model in three hospitals, with xgboost model using both vital sign and chronic illness. Bold line represents AUC before FL and transparent line represents AUC after FL. Taipei, Hsinchu and Yunlin are in red, blue and orange respectively.

The Xgboost model was initially evaluated on the Taipei cohort, achieving high predictive performance with an area under the curve (AUC) of 0.96 (Figure 4 and Table 6). However, when the pre-federated learning model was applied to other cohorts, its performance declined. After implementing federated learning, the AUC of the Taipei cohort decreased to 0.90, while the performance of other cohorts improved to meet the required standards. The decrease in Taipei cohort result from combining training results from three cohort making the model less specific to individual cohort. Whereas the increase in Hsinchu and Yunlin cohorts is because after federated learning the model has more understanding of Hsinchu and Yunlin cohorts. These results (Figure 4 and Table 6) indicate that federated learning helps to enhance the generalizability of the model across diverse cohorts, while also protecting patient privacy by ensuring data is not centralized.

**Table 6:** Comparison of AUC before and after the application of federated learning, with xgboost model using both vital sign and chronic illness.

Models	Taipei	Hsinchu	Yunlin
AUC before FL	0.96	0.82	0.81
AUC of FL	0.90	0.93	0.90

The inclusion of vital signs in conjunction with chronic illnesses improved overall model performance compared to models relying solely on chronic illness data. This demonstrates the significance of integrating diverse data types to achieve robust predictive capabilities in different patient populations.

## 4 Discussion

Our findings demonstrate that XGBoost outperformed other algorithms tested in this study for predicting 30-day mortality among hospitalized COVID-19 patients. The inclusion of vital signs significantly enhanced model performance, achieving an AUC of 0.96 (Table 3). While the results confirmed the importance of dynamic predictors such as pulse rate, oxygen saturation, blood pressure, and respiratory rate, further discussion is warranted to explore broader implications and contextualize these findings within existing clinical and research frameworks.

Machine learning has increasingly been recognized as a transformative tool in addressing complex interactions in high-dimensional clinical datasets. Traditional statistical methods, such as logistic regression and LASSO, often lack the capacity to capture nonlinear relationships and interdependencies, limiting their predictive accuracy (27, 28). Ensemble learning methods, including random forest and gradient-boosted decision trees (GBDT), have demonstrated superior performance in such scenarios (29). XGBoost, as a leading GBDT algorithm, leverages an iterative approach to optimize predictions and effectively handle missing or sparse data (30). This capability, combined with its robustness in incorporating both static and dynamic clinical variables, underscores its relevance for healthcare applications, particularly during a global health crisis like COVID-19.

A key strength of this study lies in its use of SHAP analysis to enhance model interpretability. One of the significant barriers to adopting machine learning in healthcare is the “black-box” nature of many algorithms. By quantifying the contributions of individual features to model predictions, SHAP analysis provides a transparent framework that bridges the gap between advanced computational methods and clinical decision-making. Identifying predictors such as chronic kidney disease, diabetes without complications, and hypertension allows clinicians to better understand the rationale behind predictions. This transparency not only fosters trust but also facilitates the integration of machine learning tools into routine clinical workflows, ensuring that decisions are informed and actionable.

In the early days, clinical diagnosis was purely based on physician's experiences with x-ray images and check-up data, lacking precision and efficiency (8). With the rise of ML, more models have been produced to estimate mortality and severity. According to systematic reviews in recent years (11, 12), the 4C mortality score was identified as the most promising model. The 4C Mortality Score is a risk stratification tool that predicts in-hospital mortality rate for hospitalized COVID-19 patients with eight parameters routinely available at hospital admission: age, sex, number of comorbidities, respiratory rate, peripheral oxygen saturation, level of consciousness, urea level, and C reactive protein (13). Its dataset was stratified from 260 hospitals across England, Scotland, and Wales. The 4C Score showed moderate diagnostic accuracy for mortality with derivation cohort area under the receiver operating characteristic curve (AUC) 0.79. However, it performs poorly on other cohorts, with AUC ranging 0.63∼0.73. Therefore, it cannot be implemented worldwide.

Previous clinical decision models, such as the 4C Mortality Score, have focused on common health data rather than comorbidities, lacking accuracy in assessing severity and mortality. Our research bridges this gap by adding 79 comorbidities to the prediction. We developed the Comorbidities and Clinical Indicators on the Mortality Model (CCIMM), a machine learning model that can accurately predict a patient's mortality rate within 30 days of hospitalization. We came up with two models: one comprising demographic information, vital signs upon admission, and underlying comorbidities; the other, comprising only demographic information and underlying comorbidities. The former one, with vital signs, has the highest AUC of 0.96. It can be used in the emergency room, where patients have unstable vital signs. The latter, without vital signs, has an AUC of 0.93. It can be used in outpatient conditions, where vital signs are rather stable and patients often have chronic illnesses. This model is also helpful at understanding the association between comorbidities and 30-day mortality rate. In addition, both models used federated learning to integrate data from three hospitals, resulting in models suitable for broader population needs.

This research has allowed us to gain insight into the impact of comorbidities and clinical indicators on mortality in COVID-19. This is in contrast to the 4C score, which assesses comorbidities not by the identities of the comorbidities, but by the number of comorbidities, in a categorical fashion separated into three groups- 0, 1 or ≥2. We use machine learning to deal with large amounts of data, testing different algorithms in hopes of getting the best results. CCIMrM can speed up the diagnostic agenda by being an accurate indicator of whether the patient needs antiviral medication. It can prevent hospitals from running out of medical supplies or isolation wards in devastating situations, and serve as an indicator of disease severity for patients and families. The model can also be applied to future epidemics, providing physicians with a quick and easy prediction. Furthermore, the best performing model was trained through federated learning. Our study extends the current understanding of mortality prediction for hospitalized COVID-19 patients by developing an accurate and explainable federated learning model based on comorbidities and clinical indicators.

Interestingly, the findings challenge certain assumptions in current clinical guidelines, particularly in the context of prescribing Paxlovid, which is an oral antiviral medication prescribe to patients with mild to moderate COVID-19. It can decrease the rate of severe COVID-19 or mortality with adjusted hazard ratios of 0.54 (31). While existing guidelines in Taiwan recommend Paxlovid for patients with conditions like diabetes mellitus, cerebrovascular disease, and chronic lung disease (12, 32), our study found these comorbidities to have a neutral or even protective association with 30-day mortality. This discrepancy underscores the need for continuous refinement of evidence-based guidelines using real-world data and advanced analytical techniques. For instance, while chronic kidney disease and BMI ≥30 remain high-priority risk factors, conditions like dementia and rheumatic diseases may warrant reevaluation based on the observed data trends. Additionally, the inconclusive findings regarding cancer highlight the necessity of individualized treatment plans rather than a one-size-fits-all approach to antiviral therapy.

Federated learning emerged as a critical tool in this study, addressing one of the main challenges in collaborative medical research—data privacy. By enabling the training of machine learning models across decentralized datasets, federated learning ensures that patient data remains localized while still contributing to a robust, generalizable model. This approach proved particularly effective in improving performance across external cohorts, even though it led to a slight decline in the AUC for the Taipei cohort. Such trade-offs underscore the potential of federated learning to enhance the scalability and applicability of AI solutions in diverse healthcare settings, where data-sharing restrictions often impede collaborative advancements.

## 5 Limitations

Despite its promising outcomes, the study has limitations that merit further discussion. First, the model's reliance on readily available clinical features, while advantageous for implementation, excludes other potentially valuable data sources, such as imaging and laboratory results. For example, chest x-rays and CT scans are often pivotal in assessing COVID-19 severity, and their integration into future models could significantly enhance predictive accuracy. Multimodal models that combine clinical, imaging, and laboratory data, as well as nuances about the severity of disease, would likely provide a more comprehensive understanding of patient risk profiles.

Second, the relatively small dataset focused exclusively on the Taiwanese population limits the generalizability of the findings. While federated learning mitigated some of these limitations by improving model performance in external cohorts, the demographic and clinical characteristics of the original dataset may still introduce biases. Expanding the dataset to include diverse populations from different geographic regions and healthcare systems would provide a more representative basis for training and validation. Additionally, the observed protective effects of certain comorbidities, such as diabetes and chronic lung disease, may reflect unique population-level characteristics rather than universal trends. Further studies are needed to validate these findings and investigate their underlying mechanisms.

## 6 Future directions

The potential of integrating time-series data into predictive models represents another avenue for future research. COVID-19 is characterized by rapid disease progression, making real-time monitoring of vital signs crucial for early intervention. Incorporating continuous data streams from wearable devices or bedside monitors could enable dynamic risk assessment, allowing clinicians to adjust treatment plans in real time. In addition, as COVID variants evolve and clinical practices change with time, a real-world implementation would require frequent updates to maintain satisfactory performance, especially in a federated setting. Such advancements would move machine learning from static prediction models to dynamic, context-sensitive tools that align closely with the realities of patient care.

Another critical area for exploration is the role of interpretability in enhancing clinician adoption of machine learning tools. While SHAP analysis offers valuable insights into feature importance, further efforts are needed to ensure that these explanations are presented in a manner that is intuitive and clinically relevant. For example, integrating visualizations of SHAP values into electronic health record systems could provide clinicians with real-time feedback on patient risk factors, enabling more informed decision-making. Additionally, user-centered design principles should guide the development of interfaces that present machine learning outputs in ways that align with clinicians' workflows and information needs.

The discrepancies observed between this study's findings and existing clinical guidelines also raise broader questions about how evidence is generated and translated into practice. Machine learning models offer the advantage of being data-driven, allowing for the identification of patterns that may not align with traditional clinical assumptions. However, these insights must be contextualized within the broader framework of clinical expertise and patient care priorities. For instance, while certain comorbidities may show a protective association with mortality in the study population, this does not necessarily negate their significance in other aspects of disease management. Collaborative efforts between data scientists, clinicians, and policymakers are essential to ensure that machine learning insights are translated into guidelines that are both evidence-based and clinically meaningful.

## 7 Conclusions

In conclusion, this study highlights the transformative potential of machine learning, particularly XGBoost, in predicting 30-day mortality among hospitalized COVID-19 patients. While the inclusion of vital signs significantly enhanced predictive accuracy, federated learning demonstrated its value in improving generalizability across diverse cohorts. The integration of SHAP analysis provided a transparent framework for understanding model predictions, fostering clinician trust and facilitating practical application. Future research should focus on addressing the limitations of current models by incorporating multimodal data, expanding population diversity, and enhancing real-time monitoring capabilities. By aligning advanced computational techniques with clinical expertise, machine learning has the potential to revolutionize risk stratification and treatment optimization in COVID-19 care, paving the way for more personalized and effective healthcare solutions.

## Data Availability

The datasets presented in this study can be found in online repositories. The names of the repository/repositories and accession number(s) can be found below: The dataset cannot be disclosed to public.
