# Supplementary material for: Using machine learning methods to investigate the impact of comorbidities and clinical indicators on the mortality rate of COVID-19
Source: Front Med Technol. 2025 Sep 22;7:1621158. doi: 10.3389/fmedt.2025.1621158 (PMC12497763; doi:10.3389/fmedt.2025.1621158)

Supplementary table 1: Chronic comorbidity used for machine learning model

| Cardiovascular system | Coronary artery disease , Myocardial infarction, Cerebrovascular disease, Cardiomyopathy, Valvular heart disease, Congestive heart failure, Peripheral vascular disease, Ventricular septal defect, Deep vein thrombosis, Hypertension, Aortic aneurysm |
| --- | --- |
| Pulmonsry system | Chronic lung disease, Asthma, Bronchiectasis, Tuberculosis, Chronic obstructive lung disease, Obstructive sleep apnea, Lung fibrosis, Interstitial lung disease, Pulmonary hypertension, Chronic restrictive lung disease |
| Endocrine and metabolism | DM without complications, DM with complications, Cushing syndrome, Hyperthyroidism, Panhypopituitarism, Adrenal insufficiency, Hypothyroidism, Hyperparathyroidism, ~~Hyperlipidemia~~, ~~Hyperuricemia~~ |
| Neurological system | Dementia, poliomyelitis, Hemiplegia paralysis, Epilepsy, Myasthenia gravis, bed-ridden, Traumatic brain injury, Parkinsonism |
| Renal system | Chronic kidney disease, Kidney stone, ~~Benign prostate hyperplasia~~, ESRD, polycystic kidney disease |
| Hepatobiliary system | Alcoholic liver disease, Non-alcoholic fatty liver disease, Hepatitis, Autoimmune hepatitis, Cirrhosis, Gall stone, Other chronic liver disease |
| Malignancy | Cancer, Cancer metastasis, Leukemia, Lymphoma, Multiple myeloma |
| Immune system and rheumatism | Immunocompromised, Primary immunodeficiency, Solid organ or blood stem cell transplantation, HIV, SLE, Sjogren, Rheumatoid arthritis, Ankylosing, Vasculitis, Polymyositis, Rheumatic disease |
| Psychiatric disease | Schizophrenia, Depression, Bipolar, Psychiatric disease |
| Other | Obesity, Pregnancy, Down Syndrome, Smoking, Anemia, Peptic ulcer, Bedsore |

Supplementary table 2**. Comparison of AUC before and after the application of Federated learning, with Xgboost model using only chronic illness**

|  | NTUH  (N= 2156) | Hsinchu  (N= 1170) | Yunlin  (N= 755) |
| --- | --- | --- | --- |
| AUC before FL | 0.93 | 0.58 | 0.60 |
| AUC of FL | 0.76 | 0.74 | 0.72 |

Supplementary Figure 1 provides an overview of the number of patients admitted to NTUH Taipei, NTUH Hsinchu, and NTUH Yunlin between April 2022 and October 2022. This analysis highlights the importance of integrating diverse data sources and applying federated learning to maintain robust predictive capabilities across institutions while safeguarding patient confidentiality.


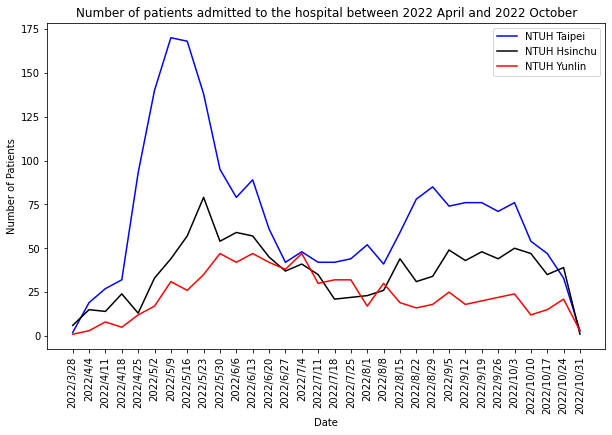


Supplementary Figure 2. Indicate the SHAP value between two features.

**
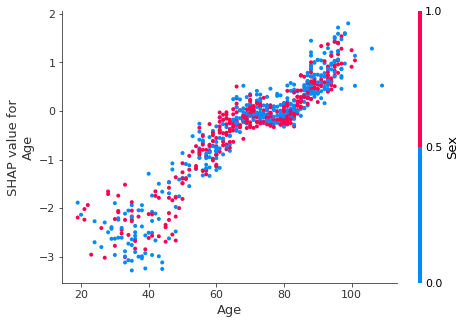

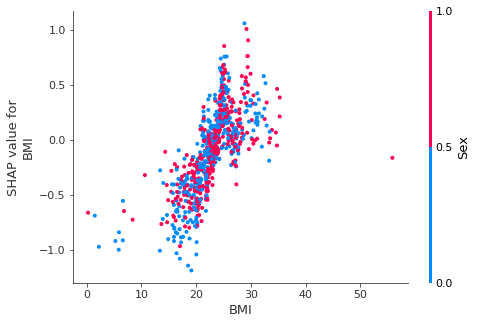

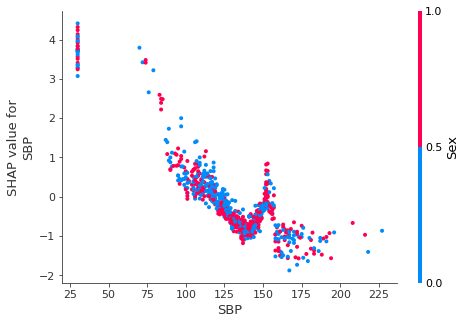

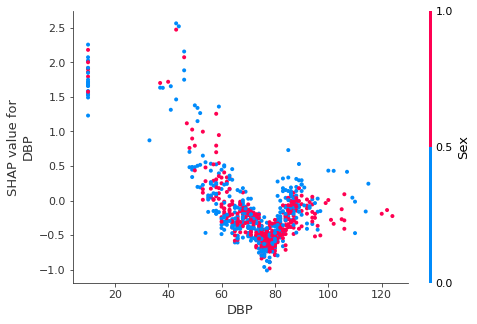

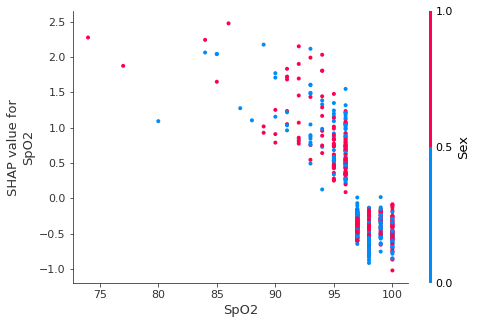

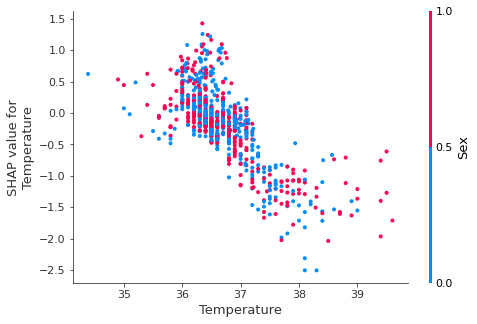

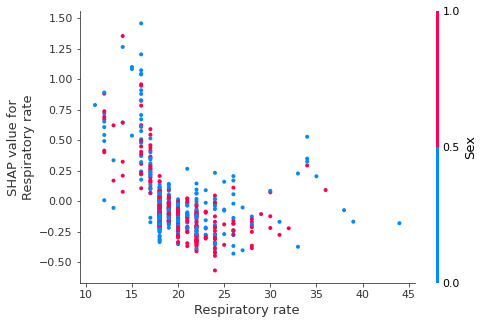

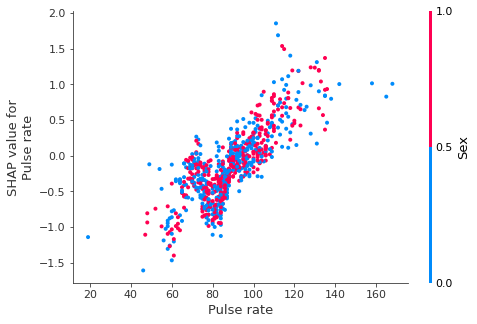
**

Supplementary Figure 3**.**Comparison of AUC of local and federated model in three hospitals, using model trained with only chronic illness


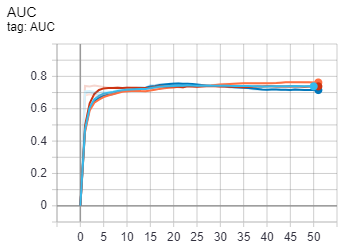

Supplement: Supplementary file 1 [file Datasheet1.docx]
